# Reverse Shoulder Arthroplasty for Primary Synovial Osteochondromatosis of the Shoulder with Massive Rotator Cuff Tear and Marked Degenerative Arthropathy

**DOI:** 10.3390/jcm7080189

**Published:** 2018-07-30

**Authors:** Toru Ichiseki, Shusuke Ueda, Daisuke Souma, Miyako Shimasaki, Yoshimichi Ueda, Norio Kawahara

**Affiliations:** 1 Department of Orthopaedic Surgery, Kanazawa Medical University, Daigaku 1-1, Uchinada, Kahoku-gun, Ishikawa 920-0293, Japan; adeu221@kanazawa-med.ac.jp (S.U.); orthoped@kanazawa-med.ac.jp (D.S.); kawa@kanazawa-med.ac.jp (N.K.); 2 Department of Pathology, Kanazawa Medical University, Daigaku 1-1, Uchinada, Kahoku-gun, Ishikawa 920-0293, Japan; miya0807@kanazawa-med.ac.jp (M.S.); z-ueda@kanazawa-med.ac.jp (Y.U.)

**Keywords:** synovial osteochondromatosis, reverse shoulder arthroplasty, massive rotator cuff tear, arthropathy

## Abstract

Synovial osteochondromatosis (SO) is a rare disease in which chondrometaplasia develops in the synovium of joints, bursa, and tendon sheaths. SO is found most frequently in the knee joint, while cases of SO developing in the shoulder joint are rare, accounting for only 1.9–5.2% of all SO cases. Moreover, most of these cases show secondary rather than primary involvement. In a patient with SO associated with extensive rotator cuff tearing and marked arthropathic changes, we performed mass resection and reverse shoulder arthroplasty (RSA), and obtained good pain relief and functional improvement. The patient was a 75-year-old woman who had developed left shoulder pain five years earlier without any known precipitating factor. The range of motion of the left shoulder showed extremely severe restriction, with flexion 80°, abduction 60°, and external rotation 0°, and prominent impingement symptoms. On plain radiographs and computed tomography (CT), prominent shoulder arthropathic changes were found. On plain magnetic resonance imaging (MRI), around the shoulder, an irregular hypointense region was identified in the center on T1-enhanced images, while hyperintense nodular lesions with a hypointense center were detected on T2-enhanced images. Since extensive rotator cuff tearing was also found, a diagnosis of OS associated with rotator cuff tearing and arthropathic changes was made. Surgery consisted of resection of a whitish mass-like floating body in the center of the joint followed by RSA. The postoperative course was uneventful, and one year postoperatively there was no recurrence of pain and the range of motion of the left shoulder had improved to flexion 140°, abduction 130°, and external rotation 30°. Moreover, no complications such as recurrence of osteochondromatosis, implant loosening, or infection were seen. On histopathological examination, the loose body was found to consist of a cartilage component and bone tissue with hyalinization. No findings indicative of malignancy were apparent, and since nodular cartilage arrangement was found, primary osteochondroma was diagnosed. These findings suggested that physical friction between the rotator cuff and the mass was the cause of the rotator cuff tearing, and that the extensive rotator cuff tearing accounted for the progression of the associated extremely severe arthropathic changes.

## 1. Introduction

Synovial osteochondromatosis (SO) is characterized by chondrometaplasia developing in the synovium of joints, bursa, and tendon sheaths, preferentially affecting young adult males. SO is considered to be a relatively rare condition and is diagnosed most frequently in the hip and elbow, followed extremely uncommonly by the shoulder, where it has a reported incidence of 1.9–5.2% of all sites [1,2,3,4,5]. Furthermore, SO is classified into primary and secondary types, with the large majority secondary, and primary SO being extremely uncommon [5]. 

In this condition, factors such as loose body incarceration promote resistance to conservative treatment in some cases, and surgical intervention becomes necessary in most. The therapy for SO of the shoulder is generally the same as that at other sites based on the Milgram classification of loose bodies in joints, with loose body resection and synovectomy performed after arthrotomy [3]. Recently, arthroscopic loose body resection has been performed more frequently, with good results reported [6,7,8]. 

We treated a case in which SO was accompanied by massive rotator cuff tearing and marked arthropathic changes, by resection of the mass and reverse shoulder arthroplasty (RSA). Since good pain relief and functional improvement were obtained, we report the details of this case here. The patient was informed that her data would be submitted for publication, and provided the appropriate consent.

## 2. Case Report

### 2.1. Case Introduction

**Figure 1:**
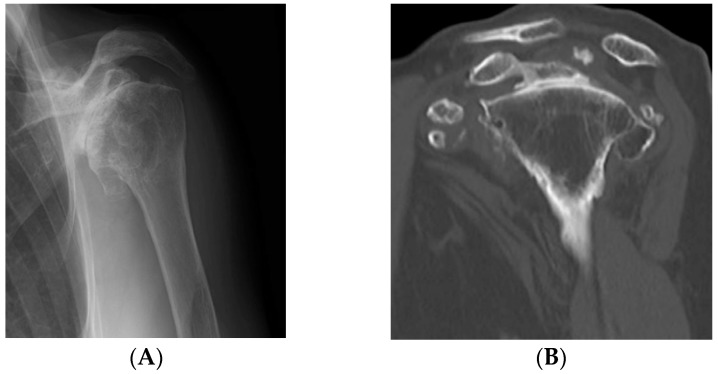

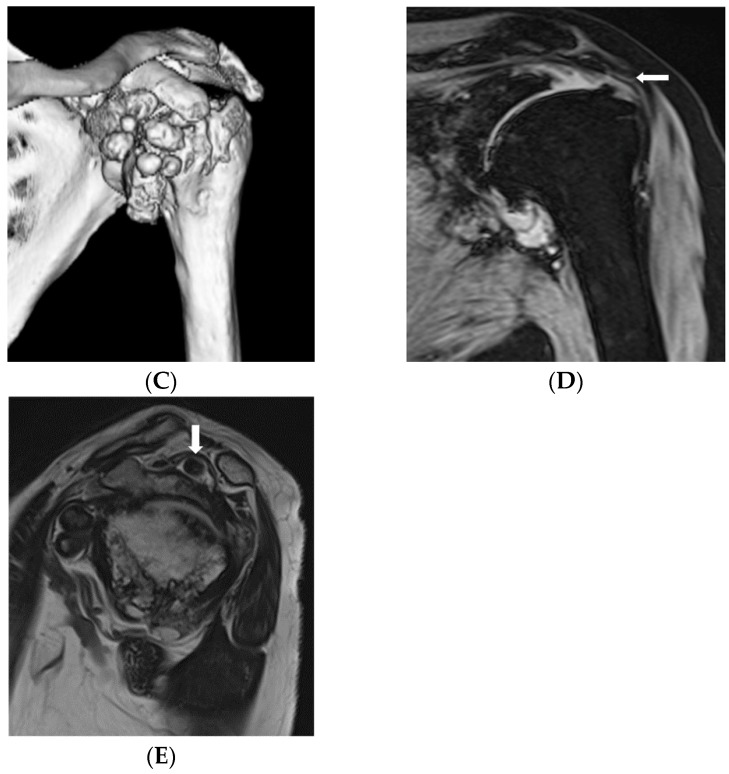
Preoperative evaluation. (**A**,**B**) On plain radiographs and computed tomography (CT), multiple well-demarcated, ossified masses were observed; (**C**) on three dimensional CT, nodular calcified lesions were similarly present; (**D**) magnetic resonance imaging (MRI) showed a massive tear of the supraspinatus tendon; (**E**) on T2-enhanced images, a hypointense mass-like lesion was found. There were many masses in the shoulder subacromium and humeral head space.

The patient was a 75-year-old woman, who had developed left shoulder pain five years earlier without any known precipitating factor. She presented to our department because of gradual difficulty in raising her left arm and worsening pain. Physical examination on presentation did not reveal any swelling or feeling of heat in the left shoulder. The range of motion of the left shoulder showed extremely severe restriction; namely, flexion 80°, abduction 60°, and external rotation 0°, and prominent impingement symptoms were found. On plain radiographs and computed tomography (CT), prominent shoulder arthropathic changes and numerous calcified lesions around the joint were found. On plain magnetic resonance imaging (MRI), around the shoulder an irregular hypointense region was identified in the center on T1-enhanced images and on T2-enhanced images (Figure 1). Routine blood examinations did not reveal any obvious abnormalities. This patient was diagnosed with synovial osteochondromatosis associated with a massive tear of the rotator cuff and shoulder arthropathic changes, for which the treatment RSA was chosen.

### 2.2. Surgical Findings

Intraoperative findings: A delto-pectoral approach was used. Full-thickness tears of the supraspinatus and infraspinatus tendons were found. When the joint capsule was incised, synovial proliferation and a whitish mass-like lesion, seemingly adherent to the synovium, were found. The intraarticular mass was resected to the extent possible, but the cartilage in the humeral head was severely damaged, while that in the glenoid cavity had almost disappeared. After loose body resection, RSA was performed. 

### 2.3. Postoperative Course

The postoperative course was uneventful, with the left shoulder pain disappearing from early in the postoperative period. At one year postoperatively, there was no recurrence of pain and the left shoulder range of motion showed improvement to flexion 140°, abduction 130°, and external rotation 30°. Moreover, no complications such as recurrence of osteochondromatosis, implant loosening, or infection were seen. 

### 2.4. Histopathological Examination

**Figure 2:**
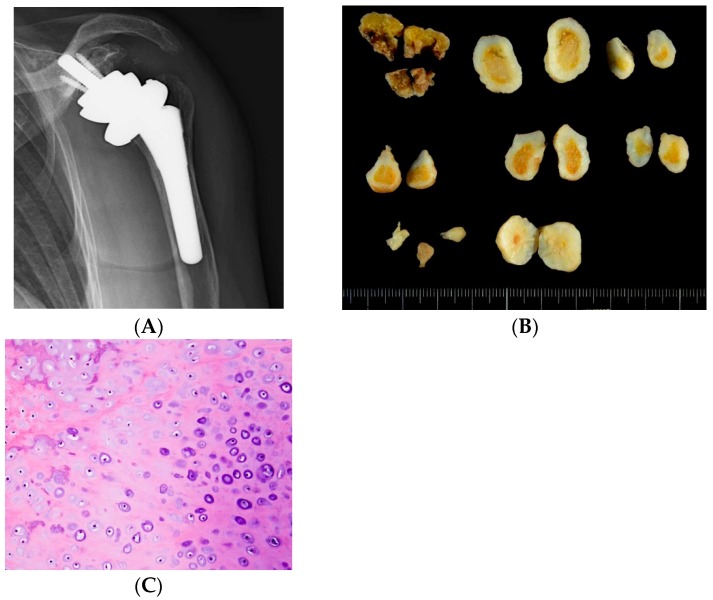
Postoperative evaluation. (**A**) Postoperative X-ray. After reverse shoulder arthroplasty, the mass in the shoulder joint has disappeared, and the course is favorable; Pathologic findings. (**B**) On a cut surface, the surface was covered with whitish cartilage, while the center consisted of yellowish bone tissue. (**C**) The histological picture showed a nodular mass covered with hyaline cartilage. In the center of the cartilage, ossification was present. No findings of malignancy were seen. Lammification structure of the hypertrophic chondrocyte was observed on the surface of the lesion, which led to the diagnosis of primary osteochondroma.

The loose body was found to consist of a cartilage component and bone tissue with hyalinization (Figure 2). No findings indicative of malignancy were evident, and since nodular cartilage arrangement was found, primary osteochondroma was diagnosed. Synovial osteochondromatosis could be diagnosed. 

## 3. Discussion

The loose bodies characteristic of this condition are easily confirmed on plain radiographs and CT when calcified or ossified, while MRI is useful in the identification of non-calcified and non-ossified foci. The definitive diagnosis is based on the pathologic findings of resected lesions. 

This condition has been classified by Milgram et al. according to the following disease stages: lesions limited to within the synovium with no loose bodies seen in the joint (Stage 1); lesions within the synovium with loose bodies seen in the joint (Stage 2); and only loose bodies in the joint seen and the synovium showing chondrometaplasia (Stage 3) [9,10,11]. The present case was judged to be Stage 2 because of the presence at surgery of inflammatory synovial proliferation and loose bodies. As a rule, the treatment for this condition is synovectomy for Stage 1, synovectomy and intraarticular loose body resection for Stage 2, and intraarticular loose body resection for Stage 3. As noted above, in cases in which arthropathic changes are absent or mild, or rotator cuff function is preserved, good results have been reported with arthroscopic mass resection and synovectomy [12,13]. Urbach et al. performed synovectomy and mass resection in five cases with SO originating in the shoulder, and reported favorable results in a 4–9 year follow-up [14]. Chillemi et al. similarly obtained postoperative early pain relief and functional recovery [15]. There are even fewer reports of cases developing rotator cuff tears against a background of shoulder SO [16,17,18], and in almost all of these mass resection alone is performed without repair or partial repair of the rotator cuff. However, in the present case, in addition to SO, both prominent arthropathic changes and massive rotator cuff tearing complicated the clinical picture. Furthermore, since severe pain was present, and the range of motion of the shoulder was also extremely restricted, RSA, in addition to loose body resection and synovectomy, was performed to obtain further functional improvement and pain relief. 

The relation between SO and progression of arthropathy remains blurred because the occurrence of SO in the shoulder is rare, precluding any clear definition of its natural course. McFarland et al. [3] reported two cases with SO that were subjected to conservative treatment for two years, although no mention was made of any arthropathy progression. On the other hand, Lunnet et al. eventually detected arthropathy in 11 of 18 cases with shoulder SO subjected to arthroscopic loose body resection and synovectomy. However, since they detected no arthropathy progression after loose body resection, they noted the possibility that progression of arthropathy may be delayed by this intervention [6]. 

In the majority of cases, SO occurs secondary to degenerative arthropathy, osteochondritis dissecans, or osteochondral fracture, with the finding of a primary lesion without any particular precipitating factor considered rare [5]. Villacin et al. [19] maintained that in the histopathological findings, nodular cartilage arrangement is present in primary lesions, while in secondary lesions due to trauma, osteochondritis dissecans, or degenerative arthropathy, laminar cartilage cell arrangement is seen. In the present case, preoperatively, SO secondary to degenerative arthropathy associated with massive rotator cuff tearing was considered present, but since histopathology confirmed the presence of nodular cartilage arrangement, the diagnosis of primary SO was thought to be more likely. 

Accordingly, we interpreted the pathophysiology of this case considering the following: (1) in the absence of any particular precipitant, primary SO occurred associated with arthropathic changes; (2) in the absence of both any history of trauma or of any particular precipitant a rotator cuff tear occurred, while a mass was present in the humeral head (shoulder subacromium), suggesting that physical friction between the rotator cuff and mass was the cause of the rotator cuff tear; and (3), that the massive rotator cuff tear accounted for the progression of the associated extremely severe arthropathic changes. 

With this experience in mind, we recommend that particular attention be paid when cases with this condition are encountered, since when a shoulder intraarticular mass is located between the subacromium and humeral head extensive rotator cuff tears may also be present. 

## 4. Conclusions

In the present case RSA for primary SO, complicated by prominent arthropathic changes and a massive rotator cuff tear, was associated with a good postoperative course. In this case, the massive rotator cuff tear occurred in the absence of any obvious precipitant such as trauma, and manifested gradually progressive pain as well as arm-raising difficulty. SO was thought to have been most likely implicated in the underlying pathophysiology of this case.
